# Using Social Media Data to Assess the Impact of Infertility on French Patients’ Quality of Life: Retrospective Observational Study

**DOI:** 10.2196/68094

**Published:** 2025-06-13

**Authors:** Mouna Mael-Ainin, Pierre-Emmanuel Bouet, Paméla Voillot, Joelle Malaab, Manissa Talmatkadi, Stéphane Schück, Nathalie Massin

**Affiliations:** 1 Ferring S.A.S, Gentilly, France; 2 Chef de service de Médecine de la Reproduction, Centre Hospitalier Universitaire, Angers, France; 3 Kap Code, 4 rue de Cléry, Paris, 75002, France, 33 144827474; 4 Hôpital Américain, Paris, France

**Keywords:** infertility, ART procedures, quality of life, social media, infodemiology, topic modeling, France, in vitro fertilization, miscarriage, assisted reproductive technology

## Abstract

**Background:**

Infertility is defined as the inability to achieve a live birth after 1 year of regular sexual intercourse, affecting 1 in 6 couples in France. The use of assisted reproductive technology (ART) for infertility issues has been steadily increasing in recent years, with in vitro fertilization being the most common type of ART. Infertility is frequently regarded as a significant life crisis for many individuals, potentially leading to depression, anxiety, social isolation, and sexual dysfunction. Couples experiencing infertility demonstrate a high prevalence of negative emotional responses and decreased life satisfaction as a result of infertility and its treatments. Social media have become key tools for finding and disseminating medical information.

**Objective:**

This study aims to explore the most discussed topics among patients with infertility and to characterize the impact of infertility and ART on their quality of life (QoL) by analyzing social media data.

**Methods:**

This retrospective observational study includes French messages from patients in France discussing their infertility between 2019 and 2022. A biterm topic model algorithm was applied to identify the topics discussed automatically. A QoL algorithm—based on the EQ-5D and SF-36 (Short Form 36 Health Survey) questionnaires—classified the messages according to 5 dimensions: physical, psychological, activity-related, social, and financial.

**Results:**

An analysis was conducted on a dataset of 26,919 messages written by 9807 patients. More than 80% (21,900/26,919) of the data were retrieved from 3 main sources: Doctissimo (9281/26,919, 34.5%), BabyCenter.fr (n=6949, 25.8%), and aufeminin.com (n=5670, 21.1%). Among the users who specified their gender (8813/9807, 89.9%), the majority were female (7942/8813, 90.1%) compared to male users (871/8813, 9.9%). The average age was 41 (SD 6.15) years. The most frequently discussed topics were the ART procedures, marked by miscarriages (5671/26,919, 21.1%), ovulation cycle monitoring (5068/26,919, 18.9%), and balancing attempts to conceive with work responsibilities (3476/26,919, 12.9%). In our study, 59% (5786/9807) of users expressed at least 1 impact on their QoL. Patients mainly reported a physical impact (3610/5786, 62.4%), particularly linked to miscarriages (2137/3610, 59.2%). The psychological impact (3020/5786, 52.2%), including fear and anxiety (1818/3015, 60.3%), was also mentioned. The social impact of infertility (n=654/5786, 11.3%)—notably its effects on the couple’s relationship (438/651, 67.3%)—the financial burden of infertility treatments (521/5786, 9%), and the impact on activities (503/5786, 8.7%) were also identified.

**Conclusions:**

Patients facing infertility share their experiences on social media. We observed a strong impact on their QoL, affecting their physical and psychological health, as well as their social, financial, and professional lives. These results underline the importance of taking into account the multiple dimensions of infertility when caring for patients and highlight the important role of social media in supporting and understanding this reality.

## Introduction

Infertility is defined as the inability to achieve a live birth after 1 year of regular sexual intercourse, affecting 1 in 6 couples [1]. The World Health Organization classifies infertility as a disease of the reproductive system, highlighting the need for increased awareness, prevention, and treatment strategies [2]. The major causes of female infertility are ovulatory dysfunction, tubal infertility, endometriosis, and uterine and cervical factors [3]. Male infertility, on the other hand, may be due to testicular dysfunction, endocrinopathies, lifestyle factors (such as obesity and tobacco use), congenital anatomical problems, gonadotoxic exposures, and aging [4]. According to the Report on the Causes of Infertility submitted to the French Ministry of Health, nearly 3.5 million people in France are impacted by infertility, and 1 in every 4 couples of childbearing age are unable to achieve pregnancy after 1 year of unprotected intercourse [5]. The global burden of infertility is increasing, influenced by demographic, environmental, and behavioral factors such as delayed childbearing [6], exposure to endocrine-disrupting chemicals [7], and rising rates of metabolic disorders such as obesity and diabetes [8]. The proposed treatments for infertility include lifestyle changes, medication, surgery, and assisted reproductive technology (ART) [9,10]. The use of ART for infertility issues has been steadily increasing in recent years [11], with in vitro fertilization (IVF) being the most common type [12]. According to the French Biomedicine Agency, the use of ART has been on the rise, with 3.6% of children, or roughly one in every 28 births, born through ART [13]. Despite ongoing research efforts, up to 25% of infertility cases remain unexplained [14].

Infertility is frequently regarded as a significant life crisis for many individuals, potentially leading to depression, anxiety, social isolation, sexual dysfunction [15], and low sleep quality [16]. Couples experiencing infertility demonstrate a high prevalence of negative emotional responses and decreased life satisfaction as a result of the condition and its treatments [17]. The psychological burden on couples substantially affects their overall well-being, willingness to continue treatment, and satisfaction with treatment outcomes [18,19].

In recent years, the rise of digital platforms and social media has provided a unique window into users’ experiences. At the start of 2024, France had 60.8 million internet users, with an internet penetration rate of 93.8%, while 50.7 million people—78.2% of the total population—were social media users [20]. Users become part of online communities and engage on forums to share their stories, seek advice, and find encouragement from others facing similar challenges [21]. Social media has been increasingly used for various health purposes—especially since the COVID-19 pandemic [22]—including sharing information and personal experiences, medical education [23], awareness around health campaigns [24,25], and online support groups [26]. Platforms such as Twitter (X Corp), Facebook (Meta), and health-related forums are increasingly used to discuss and share information about health topics [22,27]. These platforms also serve as valuable repositories of patient-generated data, offering insights into how infertility affects individuals’ lives. Females experiencing infertility reported using social media for information, solidarity, and the opportunity to receive and give support [19] and as a way to cope with the challenges associated with infertility diagnosis and treatment [28]. Males also turn to social media to share their struggles with infertility, describing feelings of emasculation and isolation to their online male infertility community [29].

While previous studies have explored the themes commonly discussed on digital platforms by individuals facing infertility, limited research has examined the impact of infertility on their quality of life (QoL) using social media data. This study aims to address this gap by exploring, for the first time, the most discussed topics among infertility patients and characterizing the impact of infertility and ART on their QoL. Using artificial intelligence techniques, including natural language processing (NLP) and machine learning (ML), this study will analyze a substantial dataset of messages from various online platforms to highlight key concerns, emotional and physical challenges, and the overall experiences of individuals facing infertility.

## Methods

### Study Design and Population

This observational, retrospective, real-world study encompassed data extracted from social media posts of patients with infertility. The duration of this study spanned from 2019 to 2022.

### Data Extraction

Messages written in French, geolocated in France, and posted between 2019 and 2022 were extracted using the Brandwatch extractor (Cision Ltd) [30]. This process involved creating a query with a list of keywords related to infertility (eg, “infertilité” [French translation for infertility], “fiv” [French translation for IVF], and “fausse couche” [French translation for miscarriage]). The complete list of the keywords included in the extraction query is provided in Multimedia Appendix 1. To perform the extraction, the Brandwatch extractor scans publicly available sources across the internet, retrieving messages that contain at least 1 keyword from the query. The extracted data included messages from public sites (eg, Twitter) and health-related forums (eg, Doctissimo). Due to restricted data access, only public Facebook pages and open groups were analyzed (ie, fiv.fr, Procréation Médicalement Assistée, Association Blog Assistance Médicale à la Procréation, Notre bataille contre l’infertilité, Avoir un enfant à 40 ans, and Fertilog), while Instagram (Instagram from Meta) and WhatsApp (WhatsApp LLC) were excluded from this study due to the complexity of their extraction (application programming interface). For the selected publicly accessible Facebook pages, data collection was performed through web crawling. All extracted posts were collected along with their associated metadata (eg, author or publication date), and compiled into Microsoft Excel (Microsoft Corp). No distinction was made in the treatment of posts obtained from different platforms, ensuring equal consideration and analysis across all sources.

### Data Processing

As the initial raw dataset contained all messages containing a keyword from the extraction query, a data cleaning process was necessary to retain only relevant posts. We therefore applied the following exclusion criteria: posts consisting of 5 words or fewer, as well as those exceeding 10,000 characters, were disregarded due to their lack of relevance and to optimize text processing. Additionally, duplicates were removed as well as sources deemed unreliable or irrelevant to our study (eg, advertising websites, forums about cars, pets, or animals). Lastly, posts not written in French were excluded.

Further filtering was conducted to identify messages written by patients and caregivers. A supervised ML algorithm was applied for this purpose. This algorithm was previously developed using a training set of 12,330 messages related to different health domains (dermatology, tobacco use, oncology, among others). The method consists of a pipeline featuring 2 Extreme Gradient Boosting (XGBoost) [31] classifiers (1 for caregivers’ experiences and 1 for patients’) applied successively. This method allowed us to identify if a post belonged to a patient, a caregiver, or neither. Evaluation of the algorithm’s performances yielded *F*_1_-scores (a measure of accuracy combining precision and recall) of 88% and 87% for the caregiver and patient classifiers, respectively.

Following these processing steps, the final “clean” dataset was obtained, consisting exclusively of posts from patients and caregivers. In the remainder of this article, we will refer to all users who are patients or caregivers as “patients.”

### Ethical Considerations

This study included data from publicly available sources; private groups or web pages were thus excluded from our data extraction process. We did not seek approval as users automatically grant their consent for the reuse of their data when they post on public platforms. Furthermore, the results of this study do not contain any identifiable information and are presented in aggregate. Information such as the name, username or handle, geographic locations, or any other sensitive data was not included. Furthermore, the names of the treatments were also removed and replaced with a generic “[TREATMENT]” placeholder in the messages.

### Data Analysis

#### Demographics

We identified patient or caregiver age and gender through a manual examination of the text, particularly when this information was explicitly provided, as exemplified below:

*[...] i'm 34 and we've been trying to have a baby for 3 years...*

In cases where age and gender information were not explicitly mentioned, we categorized the data as “undetermined.”

#### Topics of Discussion

Main discussion themes were identified through the examination of all posts from patients regarding infertility using biterm topic modeling (BTM) via the BTM *R* package. BTM is an NLP, text mining approach that analyzes large volumes of text and clusters similar text based on common topics. The model automatically groups messages into different categories—each representing a specific topic—in descending order of frequency. For each category (ie, topic), BTM provides a list of the most recurring words, helping to determine the general focus of the topic, along with the subset of messages classified under it. For example, a BTM result about breast cancer might include the following:

*Topic 1│Proportion of messages=x│List of most frequently mentioned words: body, image, confidence, scars, mastectomy, hair loss, appearance, self-esteem, femininity*.

These words suggest a focus on body image issues, which can be further validated when reviewing the subset of messages associated with topic 1. This allows us to assign a title to the topic, such as *Impact of Breast Cancer on Body Image.*

In this study, we applied BTM without prior knowledge about the topics that might emerge to ensure an unbiased analysis. As a result, BTM automatically organized the posts into categories, ranked by the frequency with which they appeared in the dataset. One message can be associated with multiple topics. Through human interpretation, these lists of words were used to label the topics, and the associated posts were thoroughly reviewed to ensure correct interpretation.

#### Impact on QoL

To assess the impact on the QoL, we developed a deep learning algorithm using regular expressions to detect relevant keywords and phrases. The algorithm categorized messages into 5 dimensions: physical (with keywords related to physical health concerns or limitations, eg, pain or motor skills), psychological (emotional distress or mental health challenges, eg fear, depression, etc), social (changes in interactions or isolation, eg, social isolation), financial (financial difficulties or work limitations, eg, debts), and daily activities (alterations in daily routines or lifestyle, eg, recreational activities and work).

The algorithm was refined and validated based on established QoL questionnaires, the EQ-5D [32] and SF-36 (Short Form 36 Health Survey) [33], to ensure accuracy and consistency in the categorization process. Subsequently, qualitative analysis was performed via the annotation of messages to validate and interpret our findings.

## Results

### Population and Posts

**Figure 1.**
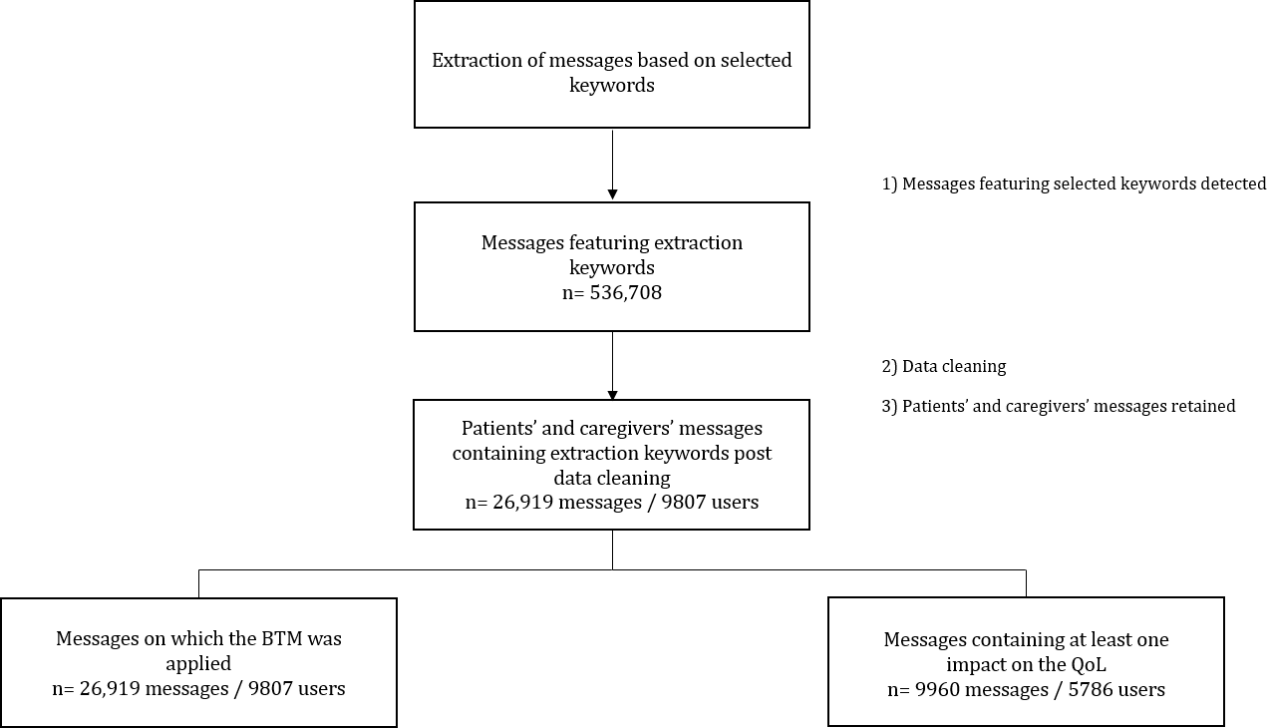
Flowchart of the data cleaning and sample selection processes, showing the number of messages (n) and users discussing infertility between 2019 and 2022. BTM: biterm topic modeling; QoL: quality of life.

Between 2019 and 2022, a total of 536,708 messages written by 141,051 internet users discussing infertility were retrieved. After data cleaning, 26,919 messages from 9807 users were retained (Figure 1).

A total of 37 sources were included in this study Multimedia Appendix 2. More than 80% (21,900/26,919) of the retrieved data was found in the following 3 sources: Doctissimo (9281/26,919, 34.5%), BabyCenter.fr (n=6949, 25.8%), and aufeminin.com (n=5670, 21.1%; Table 1). Subsequent analyses were not segmented between sources.

**Table 1.** Top 10 geolocated data sources in France for messages about infertility posted between 2019 and 2022.

Forum or social media	Posts, n (%)	Users, n (%)
Doctissimo	9281 (34.5)	1716 (17.5)
BabyCenter.fr	6949 (25.8)	3438 (35.1)
aufeminin.com	5670 (21.1)	1802 (18.4)
fiv.fr	1597 (5.9)	739 (7.5)
Twitter	1103 (4.1)	850 (8.7)
enceinte.com	911 (3.4)	335 (3.4)
Parents.fr	254 (0.9)	108 (1.1)
Facebook	231 (0.9)	216 (2.2)
HardWare.fr	224 (0.8)	134 (1.4)
Psychologies	149 (0.6)	103 (1.1)
Others	550 (2.0)	366 (3.7)

Among the users who specified their gender (8813/9807, 89.9%), the majority were female (7942/8813, 90.1%) compared to male users (871/8813, 9.9%). The gender of the rest of the users was undetermined (n=994/9807, 10.1%). The average age was 41 (SD 6.15) years.

### Topics of Discussion

Following the application of the BTM on the complete clean dataset (N=26,919), various discussion topics were identified thanks to human interpretation of each topic’s most associated terms (Table 2).

**Table 2.** List of 8 main identified topics of discussion about infertility among French internet users and their proportion of messages, with examples of messages.

Topics	Proportion of messages, n (%)	Example of message
Assisted reproductive technology procedures and miscarriages	5671 (21.1)	“Yes, don’t be too fooled by the figures: not all women produce the same amount of beta hcg. That’s why the gynecologists don’t dwell on it. I’m in ART..they say that it’s only the ultrasound that counts. I also had a miscarriage in March and I tell myself that when it’s going to work again I’m not going to be happy right away.”
Tracking ovulation cycles	5068 (18.9)	“Here I am at day 37, stillno menstruation, although it’s been almost2 years since my cycles last exceede3d1 days!”
Balancing attempts to conceive with work responsibilities	3476 (12.9)	“Yes, girls, I’m trying not to think about it too much after two miscarriages, I’m scared. What scares me is that at work I sometimes have to carry heavy things, I’m going to be careful, I can’t tell my boss, she could fire me.”
Pregnancy and ovulation testing	3446 (12.8)	“Here, I did the insemination on Monday. I’m not sure when I ovulated though. I’ve done ovulation tests, but I’m not sure how to interpret them. Since I took [TREATMENT] last Saturday, I think that influences the result. Since Monday, my tests have been dark. What do you think?”
Ultrasound scans	3142 (11.7)	“Transfer done! Now I just have to wait... I got a picture of the embryo and another of the ultrasound“
Waiting periods before pregnancy	2981 (11.1)	“We had a baby after 20 years of infertility and 12 years with my husband ... We did the mourning of having children and bam pregnant!!!”
Egg donation	1525 (5.7)	“As someone who has had to deal with repeated failures, I try to tell myself that the most important thing is to be able to bear a child one day. there’s still egg donation. I’m holding on to this idea because I know it would make me very happy.”
Pain and physical discomfort	1286 (4.8)	“Girls, has [TREATMENT] DPO 10 disappeared yet? This morning when I got up I felt pain on my right ovary like a stitch on the side, weird, has it ever happened to you?”

The discussions among patients dealing with infertility covered a range of deeply personal experiences. The most talked-about topics included ART procedures, particularly those resulting in miscarriages (5671/26,919, 21.1%). Many users discussed their struggle with multiple miscarriages, expressing the stress and anxiety of facing potential future losses. They detailed the emotional and physical toll of repeated unsuccessful attempts. Despite these challenges, some shared messages of encouragement and support, offering solidarity among women facing the same ordeal, as well as uplifting stories of ART journeys despite many obstacles. Tracking ovulation cycles was another major topic (n=5068, 18.8%). Users discussed their ovarian stimulation, describing the process, treatment, and medical check-ups. Together, they tried to better understand their cycle and recognize its signs. Balancing attempts to conceive with work responsibilities (n=3476, 12.9%) were also discussed. Many described the exhaustion they experienced during these attempts or with early-stage pregnancies, whether natural or medically induced. Some found it impossible to continue working, while others adjusted their activities—by “slowing down” or even taking a break from work—to favor implantation and reduce their risk of miscarriage. Pregnancy and ovulation tests (n=3446, 12.8%) were another recurring topic. Users turned to social media for support in interpreting their tests in case of ambiguity, shared their disappointment when tests were negative, and debated the “right time” to take them. They also discussed what was most reliable and expressed their fears about the outcomes. Updates from ultrasound scans (n=3142, 11.7%) were frequently shared, alongside conversations about the waiting period before achieving pregnancy (n=2981, 11.1%). Users also discussed the complexities surrounding egg donation (n=1525, 5.7%) and the pain and physical discomfort associated with fertility treatments and the reproductive system as a whole (n=1286, 4.8%).

### Impact on QoL

In our study, 59% of users (5786/9807) expressed at least 1 impact on their QoL.

**Figure 2.**
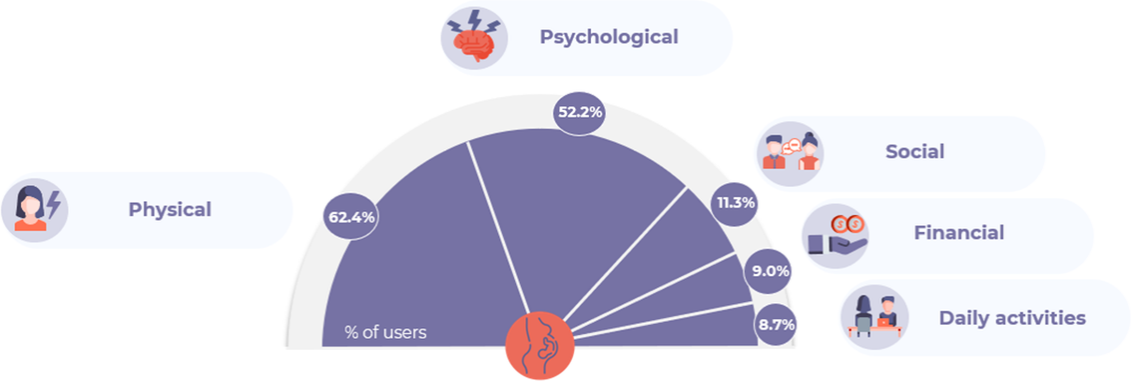
Proportions of French internet users reporting the impact of infertility on their QoL on social media between 2019 and 2022. QoL: quality of life.

Users turned to social media to describe the extent of infertility’s impact on their QoL. The most frequently reported impacts were physical (3610/5786, 62.4%), followed by psychological (n=3020, 52.2%), social (n=654, 11.3%), financial (n=521, 9.0%), and daily activities (n=503, 8.7%; Figure 2).

The physical impact (3610/5786, 62.4%) mainly included reports of miscarriages (2137/3610, 59.2%), where patients may have endured multiple miscarriages and ongoing repercussions. Menstruation and pregnancy symptoms (1375/3610, 38.1%), such as pain and nausea, and treatment side effects (552/3610, 15.3%), such as fatigue and weight gain, were also common. An example of a message shows the extent of the physical impact: “I’ve had several miscarriages and the pain never gets easier, horrible cramps and losing blood, it’s excruciating.”

The psychological impact (3020/5786, 52.2%) included feelings of fear, anxiety, and distress (1818/3015, 60.3%), such as stress over test results and postcomplication trauma (784/3015, 26%), and feelings of despair (250/3015, 8.3%). An example message:

My period is here ... a 26-day cycle. I can’t take it anymore, I’m fed up ... I never thought we’d have so much trouble getting pregnant. One more month and it’ll be a year for us trying ... I already don’t have the strength to fight any more, so how will I have the energy to go any further with the tests, to make an appointment for ART as my doctor advised?

The social impact (654/5786, 11.3%) affected couples’ relationships (438/651, 67.3%) and family dynamics (139/651, 21.4%), as shown in the following example: *“*That’s the way it was with us! Simply, I couldn’t stand any more ivf, my moral state was destroying our family, so we would either accept not having children or ask for help.”

The financial impact (521/5786, 9%) mainly revolved around treatment costs (297/518, 57.3%), which were related to users’ desire to attend reputable centers in France or abroad, leading to additional expenses, or when social security reimbursement limits were reached after multiple IVF failures. Pregnancy tests (98/518, 18.9%) were also a concern as they are nonreimbursed. The following message illustrates the financial impact:

Appointment taken at [CITY] for July 17, I had my file transferred. although I know that nothing is easy in ART but financially I’m stopping after [CITY]! Visits cost 130 euros, 50 of which I pay... 350 per hospitalization. everything is times 3! Damn, I missed a great vacation... yeah well, I’m a little angry now.

The impact on daily activities (503/5786, 8.7%) affected professional life (232/503, 46.1%), with many taking time off for appointments and exacerbated symptoms. Daily life inconveniences (143/503, 28.4%) and impacts on sexual life (76/503, 15.1%) were also noted. An example message:

Yes, the drive to the fertility specialist is complicated—1.5 hours each way if there’s no traffic, since it’s right in the middle of [CITY]. The appointment itself lasts only 15‐20 minutes, and then it’s another 1.5-hour drive back, assuming no traffic... When we go, we basically have to set aside the whole day. Right now, we’re not working, so it’s manageable, but we still have to coordinate with other appointments. Of course, cycles never go as planned—case in point, for me! 😬

## Discussion

### Principal Findings

The most discussed topics about infertility on social media included the emotional and physical challenges of ART procedures, particularly those resulting in miscarriages (5671/26,919, 21.1%). Users also described the stressful tracking of ovulation cycles (n=5068, 18.9%), balancing attempts to conceive with work responsibilities (n=3476, 12.9%), the rollercoaster of pregnancy and ovulation testing (n=3446, 12.8%), and updates from ultrasound scans (n=3142, 11.7%). Discussions also included the waiting periods before achieving pregnancy (2981, 11.1%), the complexities surrounding egg donation (n=1525, 5.7%), and the physical discomfort associated with fertility treatments (n=1286, 4.8%). In this study, 59% (5786/9807) of users expressed at least 1 impact on their QoL. In comparison, a study on individuals enduring infertility found that 74.1% reported an unsatisfactory ferti-QoL score, indicating an even higher burden [34]. Our findings highlighted that women were the most affected (7942/8813, 90.1%), which aligns with research showing that within an infertile couple, women tend to have a lower QoL than their male counterparts [35,36], facing significant impact on their QoL physically, mentally, and socially [37].

Our analysis revealed that infertility and its treatments had a significant impact on individuals’ QoL in various aspects, aligning with findings of previous research on infertility [38-42]. This study highlighted a dominating physical impact of infertility (3610/5786, 62.4%), closely followed by its psychological impact (3020/5786, 52.2%). Indeed, infertility and its treatments introduce several physical challenges. Patients undergoing fertility treatments often experience side effects such as discomfort, poor tolerance, fatigue, dizziness, abnormal menstrual bleeding, which can lead to the discontinuation of treatments [38,43]. The stress associated with infertility and its treatments can lead to feelings of anxiety, depression, and emotional distress [43]. Patients often face cycles of hope and disappointment, particularly when waiting for test results or fearing miscarriage. Failure to conceive can leave lasting emotional scars, sometimes resulting in posttraumatic stress symptoms, especially in the case of recurrent pregnancy loss [44]. Feelings of despair and hopelessness are common, especially after repeated treatment failures, leading some individuals to abandon their hopes of having children. This creates a vicious cycle, as infertility increases stress levels [45,46], and stress may, in turn, exacerbate infertility. The emotional impact of miscarriages following ART procedures is particularly devastating, with patients experiencing profound grief, sadness, and a sense of loss. The psychological toll can be compounded by feelings of failure, guilt, and loneliness [47], as well as the fear of experiencing subsequent miscarriages [48]. The physical and psychological impacts often spill into all aspects of patients’ lives. The time and mental energy required to manage this condition are immense. Managing treatments, coping with potential failures and losses, rigidly tracking ovulation cycles, and meticulously planning each decision consume significant portions of a patient’s life, leaving little time for other interests. This overwhelming burden affects everything from work responsibilities to simple leisure activities, depriving individuals of the time and space needed for a balanced life. Women enduring infertility often find it challenging to maintain high work productivity while coping with the side effects of treatments and the numerous medical examinations and procedures [49]. These treatments are often time-consuming and unpredictable, complicating the ability to manage daily life effectively [39]. Many women choose not to discuss their infertility treatments at work, but this can become complicated, especially when increased absenteeism due to medical appointments or managing treatment side effects becomes necessary [49]. The amount of time dedicated to the management of infertility limits patients’ ability to engage in simple leisure activities, hobbies, and self-care [39,50]. A study found that over 18 months, the average time spent on fertility care was 125 hours, equating to approximately 15.6 days, assuming an 8-hour workday [51]. This raises concerns about the potential impact on their psychological state, as the constant stress and lack of respite can be overwhelming. Furthermore, the absence of time for their usual activities and hobbies may cause their identity to become solely defined by their infertility struggles. These significant impacts inevitably extend to affect patients’ relationships. The constant stress can strain the couple’s life, and their sexual relationship may also suffer as a result [41,52]. Sexual intercourse becomes unenjoyable and only a means to conceive [39]. Some women express feelings of anger and resentment toward their partner, particularly when their partner is identified as the cause of infertility. Additionally, they may perceive them as being less invested and more passive in the journey to conceive, leading to further emotional distress and tension within the relationship [39]. What exacerbates the situation is the social stigmatization that couples, particularly women, might face, and the pressure from their social environment. This often leads them to conceal their condition, and avoid social gatherings and events related to babies, such as baby showers and children’s birthdays. Consequently, their relationships with family members may suffer, potentially leading to social isolation and feelings of shame [52-57]. On a financial level, patients reported treatment costs as a significant concern. This is particularly the case of repeated IVF failures, as IVF is only reimbursed up to 4 cycles [58]. Despite comprehensive health insurance in France, research found an increased risk of nonaccess among women below the poverty line and populations living in remote areas far from fertility centers [59]. Due to limited access to oocyte donation in France, derived from both a lack of donors and a shortage of clinics offering the service, France is 1 of the top 4 sources of patients seeking cross-border reproductive care in Europe [60,61]. Moreover, the lower cost of treatment, which can be found in other countries, such as Greece, is another motivating factor [62]. Indeed, a study revealed that 91% (n=22) of participants traveled to Greece, with cost being an important factor in their decision [63]. However, some women with the financial means opt for cross-border fertility treatments due to the limited access to oocyte donation and difficulty in accessing ART in France, including oocyte and sperm donation. They may turn to countries such as Spain and Belgium [63], which may exacerbate financial costs.

Given the profound impact of infertility on patients’ lives, as well the possible secrecy and stigma surrounding this situation concerning their social environment, many patients find solace and support in online communities. Social media can offer information [64,65], as well as the ability for patients to connect with other people with similar experiences [65]. The ability to engage anonymously in online forums is highlighted as one of the most appreciated aspects [65]. Numerous women indicate a preference for this anonymity, as it enables them to express their feelings and find resources to support their psychological well-being without needing to reach out to those around them. This also allows them to maintain a sense of human connection [65,66]. Recognizing the emotional burden that infertility imposes, ART services in France have also introduced several measures to help both patients and professionals better manage the psychological impact of infertility and its treatments. Many clinics now integrate psychological counseling into their fertility treatment protocols. This is particularly beneficial given that French patients are more likely to require support from a mental health professional due to fertility problems [67]. Engaging in therapy can help couples alleviate emotional distress, gain fresh perspectives, and expand their range of actions, instead of succumbing to unrealistic expectations, blame, guilt, resignation, and hopelessness [68]. Additionally, guidelines for mental health specialists for counseling and training of medical staff are increasingly being developed to help couples navigate their infertility journeys more effectively [68,69].

### Limitations

This study includes limitations that are common in social media research. The data were derived from publicly available social media sources; private sources such as closed Facebook groups and WhatsApp were thus excluded. Social media data is based on patient-provided information, so in cases where information was lacking, context and representativeness became uncertain. Our study included a potential recall bias as it was based on users’ self-reported data, their memory, and subjective interpretation. Additionally, individuals posting on social media may be of certain socioeconomic backgrounds and literacy capacities, which may affect the representativeness of our findings. We are unable to verify users’ characteristics, such as demographics and clinical information, which could limit the accuracy of testimonials. Moreover, it is worth noting that relevant posts might have been mistakenly eliminated during the filtering process. To reduce background noise, we used threshold values similar to our previous work [70-72], which may have constrained the natural processing analysis. Despite these limitations, this study provides valuable insights into the impact of infertility on patients’ lives, derived directly from their input.

### Conclusions

The impact of infertility on patients’ lives—physically, psychologically, socially, financially, and in their daily activities—is undeniable. This study revealed the challenges faced by individuals struggling with infertility, with miscarriages being their main reported concern. Online platforms play an important role in engaging patients to narrate their experiences, seek help, and find solace while receiving the requisite support. Given the significant toll of infertility, health care providers can use these insights to develop more empathetic and personalized patient care, with a focus on psychological support to address the emotional and social challenges that patients might face. Future research could further use ML and NLP to delve deeper into patient concerns and the discussions around infertility. These social media discussions can be used by clinicians to improve the QoL and treatment outcomes of individuals dealing with infertility.

## Supplementary material

10.2196/68094 Multimedia Appendix 1 The extraction query.

10.2196/68094 Multimedia Appendix 2 Sources were included in this study.

## Abbreviations

ART: assisted reproductive technology
BTM: biterm topic model
IVF: in vitro fertilization
ML: machine learning
NLP: natural language processing
QoL: quality of life
SF-36: Short Form 36 Health Survey
XGBoost: Extreme Gradient Boosting
