# Supplementary material for: Using Social Media Data to Assess the Impact of Infertility on French Patients’ Quality of Life: Retrospective Observational Study
Source: J Med Internet Res. 2025 Jun 13;27:e68094. doi: 10.2196/68094 (PMC12180682; doi:10.2196/68094)
Supplement: Multimedia Appendix 1 [file jmir-v27-e68094-s001.docx]

((baisse OR probleme OR trouble) NEAR/10f fertilite)

OR ((desir OR difficulte) NEAR/5f grossesse)

OR (difficulte NEAR/3f enceinte)

OR ((difficulte NEAR/3f (concevoir OR conception)) NEAR/20 (bebe OR grossesse OR maman OR parent OR medecin OR gygy OR gyneco OR progesterone OR pma OR enfant OR insemination OR uterus))

OR "projet parental" OR "fausse couche"

OR infertilite OR infertillite

OR ("pma" NEAR/30 (bebe OR "bb" OR grossesse OR enceinte OR enfant OR hcg OR gygy OR medecin OR gyneco* OR infertilite OR uterus OR maman OR conjoint))

OR "hcg" OR ovitrelle OR ovalep OR gonal-f OR clomid OR pregnyl OR menopur

OR (ovulation)

OR ((stimulation OR insuffisance) NEAR/5 ovarienne)

OR (taux NEAR/3f (grossesse OR accouchement OR amh))

OR "fiv" OR insemination OR gonadotrophine OR "icsi"

OR (don NEAR/3 (ovocyte* OR sperm*))

OR (transfert NEAR/5 embryon)

OR (bioethique NEAR/10 (grossesse OR PMA OR FIV OR infertilite))

NOT (chien OR chat OR minette OR mouton OR "ivg" OR "contraception urgence"~3 OR norlevo OR Levonorgestrel OR ellaone OR avorte* OR musculation OR  Britney OR "Karine ferri" OR pologne OR steroide OR "pc" OR (jeu* NEAR/3f (ligne OR video OR online)) OR (acheter NEAR/3 ligne) OR ((anti OR contre OR opposant) NEAR/3f "pma") OR  site:1001cocktails.com OR site:openclassrooms.com OR site:cbanque.com)
