# Supplementary material for: Using Social Media Data to Assess the Impact of Infertility on French Patients’ Quality of Life: Retrospective Observational Study
Source: J Med Internet Res. 2025 Jun 13;27:e68094. doi: 10.2196/68094 (PMC12180682; doi:10.2196/68094)
Supplement: Multimedia Appendix 2 [file jmir-v27-e68094-s002.docx]

| **Forum/Social Media** | **Number of posts** | **Number of users** |
| --- | --- | --- |
| Doctissimo | 9281 | 1716 |
| babycenter.fr | 6949 | 3438 |
| Au Feminin | 5670 | 1802 |
| fiv.fr | 1597 | 739 |
| X | 1103 | 850 |
| enceinte.com | 911 | 335 |
| parents.fr | 254 | 108 |
| facebook | 231 | 216 |
| hardware.fr | 224 | 134 |
| psychologies | 149 | 103 |
| Magic maman | 148 | 85 |
| Journal des femmes | 112 | 74 |
| beauté test | 78 | 26 |
| madmoizelle.com | 68 | 53 |
| alexia.fr | 29 | 29 |
| carenity.com | 20 | 20 |
| futura-sciences | 14 | 13 |
| Ligue contre le cancer | 14 | 10 |
| journaldesfemmes.com | 12 | 12 |
| babyfrance.com | 9 | 3 |
| Yabiladi | 9 | 9 |
| lescigognesdelespoir.com | 8 | 3 |
| jeunepapa.com | 5 | 5 |
| Onmeda | 5 | 5 |
| la-sclerose-en-plaques | 3 | 3 |
| famili.fr | 2 | 2 |
| lacoccinelle.net | 2 | 2 |
| Lymphome espoir | 2 | 2 |
| MeaMedica | 2 | 2 |
| etreenceinte.com | 1 | 1 |
| Forum ados/public.fr | 1 | 1 |
| infobebes.com | 1 | 1 |
| lesimpatientes | 1 | 1 |
| migraine-solution.fr | 1 | 1 |
| pcmfrance.com | 1 | 1 |
| racontezvosreves.com | 1 | 1 |
| ruche-apiculture.com | 1 | 1 |
